# Supplementary material for: The Small RNA Universe of Capitella teleta
Source: Front Mol Biosci. 2022 Feb 25;9:802814. doi: 10.3389/fmolb.2022.802814 (PMC8915122; doi:10.3389/fmolb.2022.802814)
Supplement: Supplementary file 1 [file DataSheet1.ZIP › Supplement/confident/CAPTEscaffold_8851_42498.pdf]

Provisional ID	:	CAPTEscaffold_8851_42498
Score total	:	188.6
Score for star read(s)	:	3.9
Score for read counts	:	181.6
Score for mfe	:	2.1
Score for randfold	:	1.6
Score for cons. seed	:	-0.6
Total read count	:	368
Mature read count	:	308
Loop read count	:	0
Star read count	:	60

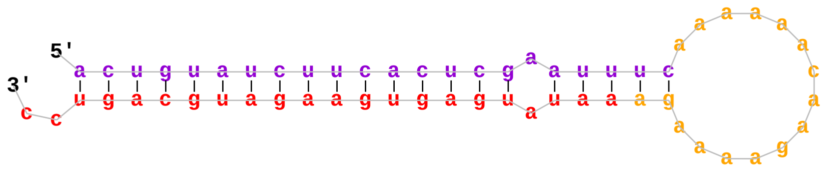

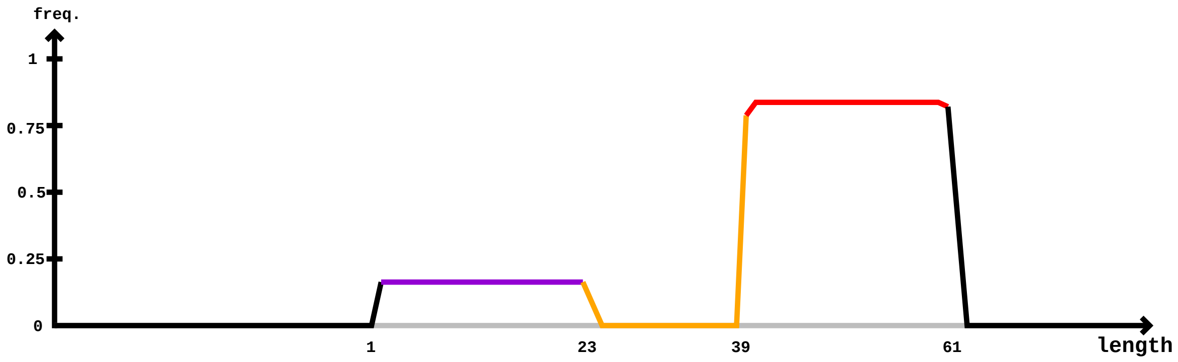

Star Mature

```
5'- ucuccaccguuguuguucucgcguggaaauggacuguaucuucacucgaauuucaaaaaacaagaaaagaaauaugagugaagaugcaguccauuuccauuguuuauacguc  -3'  obs
    ucuccaccguuguuguucucgcguggaaauggacuguaucuucacucgaauuucaaaaaacaagaaaagaaauaugagugaagaugcaguccauuuccauuguuuauacguc       exp
    ..............((....((((((((((((((((((((((((((((.(((((..............))))).))))))))))))))))))))))))).)))....))...   reads  mm  sample
    ................................acugAaucuucacucgaauuuc..........................................................   1      1   seq
    ................................acuguaucuucacCcgaauuuc..........................................................   1      1   seq
    ................................acuguaucuucacucgaauuuc..........................................................   57     0   seq
    ................................Gcuguaucuucacucgaauuuc..........................................................   1      1   seq
    ......................................................................aauaugagugaagaugcaguc.....................   6      0   seq
    ......................................................................aauaugagugaagaCgcagucc....................   1      1   seq
    ......................................................................aauaugagGgaagaugcagucc....................   1      1   seq
    ......................................................................aauauAagugaagaugcagucc....................   1      1   seq
    ......................................................................aauaugagugaagaugcagucc....................   275    0   seq
    ......................................................................aauaugagugaagaugcaAucc....................   1      1   seq
    ......................................................................aauGugagugaagaugcagucc....................   1      1   seq
    ......................................................................aauaugagugaagaugcagCcc....................   1      1   seq
    ......................................................................aauaugagugaagaugcaguccU...................   3      1   seq
    .......................................................................auauAagugaagaugcagucc....................   1      1   seq
    .......................................................................auaugagugaagaugcagucc....................   17     0   seq
```
